# Analysis of multiple factors involved in acute progressive cerebral infarction and extra- and intracranial arterial lesions

**DOI:** 10.3892/etm.2014.1624

**Published:** 2014-03-14

**Authors:** YUEFU CHEN, YAJIE LIU, CHENGHONG LUO, WEIHENG LU, BINRU SU

**Affiliations:** 1 Department of Neurology, Shilong People’s Hospital, Southern Medical University, Dongguan, Guangdong 523326, P.R. China; 2 Department of Neurology, Zhujiang Hospital, Southern Medical University, Guangzhou, Guangdong 510282, P.R. China

**Keywords:** progressive cerebral infarction, carotid atherosclerosis, cerebral artery stenosis, stroke in progression

## Abstract

In order to identify the potential factors involved in the development of acute progressive cerebral infarction (PCI), the association between potential risk factors and extra- and intracranial arterial lesions was investigated. A total of 608 patients underwent cerebral angiography to analyze the morphological characteristics between the PCI and NPCI groups. In addition, data from numerous cases of extra- and intracranial arterial lesions were collected and compared with the control groups, and the associations between the severity of arterial lesions and the potential influential factors were analyzed. In the blood vessels responsible for cerebral infarction, various degrees of atherosclerotic plaques and stenosis were observed. Age, high-density lipoprotein (HDL) levels, glycosylated hemoglobin and blood pressure affected the degrees of hardening, plaques and stenosis. Analysis of cerebral artery stenosis revealed that age, diabetes mellitus and plasma fibrinogen were risk factors for cerebral artery stenosis, while the HDL/low density lipoprotein ratio was a protective factor. Therefore, the results of the present study indicate that the lesions of blood vessels are a major pathological change in PCI and multiple factors are involved in the pathogenesis.

## Introduction

Progressive cerebral infarction (PCI) is a brain disorder caused by insufficient blood supply. Cerebral infarction may lead to cerebral ischemia, hypoxia, necrosis and finally neurological deficit (1). Recently, studies of cerebral infarction have focused on identifying risk factors. Multiple measures have also been taken, including health education, acute-stage patient care, vascular stenting and surgery, neuroradiology, early rehabilitation. However, the prevention and treatment of cerebral infarction remains largely unsuccessful and the prognosis is severe. Between 50 and 70% of surviving patients are left with paralysis, aphasia and dementia (2). Therefore, it is particularly important to identify risk factors for the development of PCI.

At present, there are no ideal strategies that effectively prevent the progression of cerebral infarction. With the rapid progress in the treatment of intravascular hydrocephalus and the continuous improvement of interventional equipment, stenting and angioplasty are feasible in the treatment of intracranial vascular stenosis (3). These treatments are recommended for patients with intracranial arterial stenosis who do not respond well to medical treatment or whose arterial stenosis is >50%, according to the guidelines from the American Society of Interventional and Therapeutic Neuroradiology, the American Society of Interventional Radiology and the American Society of Neuroradiology (4). However, the clinical value of vascular balloon angioplasty and stent implantation in preventing the progression of cerebral infarction remains unknown.

The present study enrolled patients with PCI and those with non-progressive cerebral infarction (NPCI) in order to compare and analyze the cerebral angiographic characteristics. Differences in vascular stenosis and vascular morphology were revealed by cerebral angiography. The aim of the present study was to provide further theoretical basis for interventional therapy of cerebrovascular disease.

## Materials and methods

### Case selection criteria

Data from 608 PCI patients (male 419, female 189) admitted to the Department of Internal Medicine at Shilong People’s Hospital (Guangdong, China) were collected between May 2010 and May 2013. The inclusion criteria met the diagnostic criteria set in the first edition of the Chinese Guidelines for Cerebrovascular Disease Prevention (5) and were confirmed by head computed tomography or magnetic resonance imaging examinations. The patients were divided into two groups: PCI and NPCI groups. The PCI group included patients who had been admitted within 24 h after the onset of the disease, but had not been treated within 6 h of onset. The diseases were progressing and the patients scored ≥2 points according to the United States National Institute of Health Stroke Scale (NIHSS) (6). The NPCI group (control group) included patients who were admitted within 24 h after onset and whose diseases had reached the peak, thus progression had stopped 6 h after onset. These patients scored <2 points on the NIHSS. The study protocol was approved by the Institutional Ethical Committee for Research on Human Subjects (Guangzhou, China) and informed written consent was obtained from each patient.

### Carotid artery ultrasonography

To examine the extent of common carotid artery atherosclerosis, the intima media thickness (IMT) and the vessel diameters were measured by ultrasonography. The severity of the carotid artery lesions was classified into four groups: Normal (IMT ≤0.9 mm), hardening (0.9 mm<IMT≤1.5 mm), plaque formation (IMT>1.5 mm) and stenosis (narrowing, >30%).

### Cerebral angiography

A Seldinger puncture was created in the femoral arteries of patients from the two groups. Angiography was performed using catheters at the aortic arch, bilateral common carotid arteries and vertebral arteries. Based on the North America Symptomatic Carotid Endarterectomy Trial method (7), vascular stenosis was assessed by doctors experienced in neurointervention. The degree of cerebral artery stenosis was classified into two groups based on the reduction in vessel diameter: Mild stenosis (≤50%) and severe stenosis (>50%). The severity of arterial lesions was evaluated.

### Statistical analysis

Continuous data are presented as mean ± SD and were analyzed with a Student’s t-test or one-way analysis of variance (when the variance was irregular Welch correction was used). Categorical data were analyzed with a χ^2^ test. To identify the potential risk factors for the development of PCI, the linear regression method and multivariate logistic regression analyses were used. P<0.05 was considered to indicate a statistically significant difference.

## Results

### Single factor analysis

Associations between multiple potential risk factors and carotid artery atherosclerosis were firstly analyzed. As shown in Table I, the incidence rate of diabetes was significantly higher in patients with carotid artery atherosclerosis when compared with those with normal carotid arteries (χ^2^=18.988; P<0.01). As the severity of atherosclerosis increased, the diabetes incidence also increased, indicating the involvement of diabetes in the pathogenesis of carotid artery atherosclerosis. Similarly to diabetes, the incidence of hypertension was also significantly higher in patients with carotid artery atherosclerosis when compared with those with normal carotid arteries (χ^2^=82.107; P<0.01). The incidence of hypertension increased to 90% in patients with carotid artery stenosis, demonstrating the effect of hypertension on carotid artery stenosis. Hyperlipidemia was more common among patients with impaired carotid arteries, despite the less evident difference in the incidence (χ^2^=10.312; P=0.016). Notably, other factors, including smoking, alcohol consumption, cholesterol and lipoprotein, were not significantly different between the normal carotid artery and the dysfunctional carotid artery groups. The ages of the patients varied among the groups with different carotid artery lesions. Stenosis occurred more frequently in older patients.

In addition, whether the factors listed in Table I contributed to the development of cerebral artery stenosis was investigated. As shown in Table II, diabetes, hypertension, hyperlipidemia and age were associated with the severity of cerebral artery stenosis, exhibiting a similar pattern to carotid artery atherosclerosis. However, smoking and alcohol consumption was also demonstrated to affect the narrowing of cerebral arteries, contrary to carotid arteries.

Furthermore, whether these factors were involved in the development of neurological deficits, including stroke and progressive stroke, was investigated. The factors that contributed to the severity of cerebral stenosis (Table II) also affected the incidence of stroke (Table III) in a similar manner. This observation also enhances the correlation of stroke and cerebral artery stenosis. However, the data showed that only hyperlipidemia, alcohol consumption and age were significantly different between the patients with or without progressive stroke (Table IV; P<0.05).

### Multivariate logistic regression analysis

Multiple linear regression analysis revealed that multicollinearity existed between systolic and diastolic pressure, total cholesterol and LDL. Due to extensive variance, two factors (diastolic blood pressure and total cholesterol) were rejected in the model and the results are shown in Table V. The results demonstrated that these factors exhibited significant differences at various levels of carotid artery atherosclerosis (hardened, hardened plaque and stenosis groups), when compared with the normal group. The odds ratio was set at >1 for risk factors and otherwise protective factors. As shown in Table VI, the influencing factors of cerebral artery stenosis included age, diabetes, plasma fibrinogen and HLR (HDL/LDL ratio), among which age, diabetes mellitus and plasma fibrinogen were identified as risk factors, whereas HLR was a protective factor. As shown in Table VII, risk factors were also identified for stroke. These included fasting blood glucose and smoking. However, multivariate analysis of the bivariate correlation between progressive stroke and cerebral artery atherosclerosis exhibited no significant correlation (Table VIII). For effects of fasting blood glucose and plasma fibrinogen (FIB) classification, as shown in Table IX, age, diabetes and smoking were important factors for carotid atherosclerosis. Similarly, age and diabetes were also the important factors in FIB classification of cerebral artery stenosis (Table X).

## Discussion

PCI is a refractory cerebral vascular disease with an incidence rate of 20–30% in patients with cerebral infarction. PCI often leads to brain deterioration and thereby significantly increases the mortality rate (8–10). The occurrence and development of PCI are affected by a number of factors and mechanisms. Among the numerous risk factors, atherosclerosis, stenosis or occlusion of the trunk and main branches of cerebral arteries are major independent risk factors (11). Consistent with a previous study (12), the data of the present study demonstrated that the corresponding vessels in the infarction region had various degrees of vascular sclerosis and stenosis.

Multiple mechanisms of cerebral infarction caused by atherosclerosis have been proposed, including intravascular thrombosis, vascular stenosis and reduced perfusion pressure in terminal cerebral vessels (8). When intravascular plaques detach from the arterial thrombus or atherosclerosis directly involves the perforator vessels, cerebral infarction may occur. The atherosclerotic vessels are more prone to thrombosis, which aggravates the preexisting vascular stenosis or occlusion (13). Thrombosis may exacerbate cerebral ischemia unless collateral circulation is formed in time. When collateral circulation does not form, cerebral infarction becomes progressive. Stenosis of the cerebral vessels is an additional mechanism underlying the progression of infarction (14). The narrowed cerebral vessels are more likely to have local thrombosis. Thrombosis may extend to the distal vessels resulting in stenosis, and detachment of the thrombus from the wall may also cause an arterial embolism (15,16). When stenosis occurs in the internal carotid, vertebral basilar or other medium-sized arteries, blood flow to the distal branches decreases. With low perfusion, the distal narrowed vessels fail to form effective collateral circulation to bypass the blockage. The results of the present study revealed that atherosclerotic plaques and stenosis existed in the corresponding vessels of cerebral infarction. These observations indicate that cerebral vascular lesions play an important role in the pathogenesis of PCI. In addition, the current study identified that numerous factors, including age, HDL, glycosylated hemoglobin and blood parameters, correlated with the severity of atherosclerosis, plaque formation and stenosis in the carotid artery. Factors affecting cerebral artery stenosis were also identified, including age, diabetes, plasma fibrinogen and HLR, among which age, diabetes mellitus and plasma fibrinogen were risk factors, while HLR was a protective factor. Therefore, in patients with acute cerebral infarction, early treatment of vascular stenosis and cerebral artery recanalization may improve cerebral perfusion, thus, prevent the progression and recurrence of cerebral infarction. A previous study demonstrated that placing a stent in the narrowed vessels of patients with PCI, particularly in those with artery stenosis when performed within 16 h of disease onset and treated within 8 h, achieves favorable effects (17). In conclusion, the results of the present study indicate that the lesions of responsible blood vessels play an important role in PCI. The observations provide supporting evidence for interventional therapy for cerebrovascular disease.

## Figures and Tables

**Table I:** Association of potential risk factors with severity of carotid atherosclerosis.

Risk factor	Carotid atherosclerosis				Total	Test statistic	P-value

	Normal	Hardening	Plaque	Stenosis			
Gender, n (%)							
Male	60 (69.0)	67 (67.7)	197 (69.6)	62 (74.7)	386 (69.9)	1.189	0.756
Female	27 (31.0)	32 (32.3)	86 (30.4)	21 (25.3)	166 (30.1)		
Diabetes, n (%)							
No	75 (86.2)	75 (75.8)	185 (65.4)	50 (60.2)	385 (69.7)	18.988	<0.01
Yes	12 (13.8)	24 (24.2)	98 (34.6)	33 (39.8)	167 (30.3)		
Hypertension, n (%)							
No	49 (56.3)	24 (24.2)	37 (13.1)	8 (9.6)	118 (21.4)	82.107	<0.01
Yes	38 (43.7)	75 (75.8)	246 (86.9)	75 (90.4)	434 (78.6)		
Hyperlipidemia, n (%)							
No	56 (64.4)	51 (51.5)	127 (45.0)	39 (47.0)	273 (49.5)	10.312	0.016
Yes	31 (35.6)	48 (48.5)	155 (55.0)	44 (53.0)	278 (50.5)		
Smoking, n (%)							
No	60 (69.0)	58 (58.6)	172 (60.8)	43 (51.8)	333 (60.3)	5.379	0.146
Yes	27 (31.0)	41 (41.4)	111 (39.2)	40 (48.2)	219 (39.7)		
Wine consumption, n (%)							
No	78 (89.7)	87 (87.9)	241 (85.2)	71 (85.5)	477 (86.4)	1.393	0.707
Yes	9 (10.3)	12 (12.1)	42 (14.8)	12 (14.5)	75 (13.6)		
Age, years^a^	53.2±11.6	62.3±11.3	66.4±10.0	68.6±8.3		43.4	<0.01
Systolic blood pressure, mmHg^a^	136.3±21.3	151.3±23.8	153.2±23.5	153.6±22.1		12.9	<0.01
Diastolic blood pressure, mmHg^a^	81.2±12.8	85.3±13.3	85.2±12.3	83.7±9.1		2.6	0.049
Platelet count (10^9^/l)^a^	235.788±67.761	253.153±95.158	237.735±76.974	236.602±70.103		1.135	0.334
International normalized ratio^a^	1.001±0.080	1.006±0.088	1.033±0.109	1.041±0.170		2.911	0.034
Plasma fibrinogen (g/l) a	3.301±0.789	3.564±0.796	3.805±0.990	3.862±1.011		7.297	<0.01
Total cholesterol (mmol/l)^a^	4.675±1.208	4.959±1.137	4.980±1.156	5.043±1.499		1.639	0.179
Triglyceride (mmol/l)^a^	1.661±1.165	1.741±1.112	1.738±1.069	1.643±0.937		0.254	0.859
HDL (mmol/l)^a^	1.087±0.334	1.226±0.508	1.135±0.549	1.114±0.340		1.451	0.227
LDL (mmol/l)^a^	2.596±0.992	2.905±0.993	2.773±1.002	2.936±1.123		2.039	0.107
HLR^a^	0.480±0.253	0.492±0.405	0.478±0.330	0.451±0.397		0.219	0.883
Fasting blood glucose (mmol/l)^a^	5.878±1.691	6.222±2.006	6.698±3.055	6.866±3.622		2.640	0.049
Glycated hemoglobin (%)^a^	6.233±1.948	6.197±1.415	6.942±2.016	6.970±1.881		4.122	0.007
Homocysteine (μmol/l)^a^	13.574±5.586	13.941±4.898	14.849±6.470	15.002±5.258		1.031	0.379

a Mean ± SD.

HDL, high-density lipoprotein; LDL, low-density lipoprotein; HLR, HDL/LDL ratio.

**Table II:** Association between potential risk factors with the severity of cerebral artery stenosis.

	Cerebral artery stenosis				
				
Risk factor	Narrowing ≤50%	Narrowing >50%	Total	Test statistic	P-value
Gender, n (%)					
Male	185 (68.0)	234 (69.6)	419 (68.9)	0.186	0.666
Female	87 (32.0)	102 (30.4)	189 (31.1)		
Diabetes, n (%)					
No	221 (81.3)	209 (62.2)	430 (70.7)	26.339	<0.001
Yes	51 (18.8)	127 (37.8)	178 (29.3)		
Hypertension, n (%)					
No	79 (29.0)	50 (14.9)	129 (21.2)	18.039	<0.001
Yes	193 (71.0)	286 (85.1)	479 (78.8)		
Hyperlipidemia, n (%)					
No	154 (56.6)	149 (44.5)	303 (49.9)	8.850	0.003
Yes	118 (43.4)	186 (55.5)	304 (50.1)		
Smoking, n (%)					
No	185 (68.0)	195 (58.0)	380 (62.5)	6.387	0.011
Yes	87 (32.0)	141 (42.0)	228 (37.5)		
Wine consumption, n (%)					
No	246 (90.4)	281 (83.6)	527 (86.7)	6.037	0.014
Yes	26 (9.6)	55 (16.4)	81 (13.3)		
Age, years^a^	60.7±12.1	65.8±10.4		−5.6	<0.01
Systolic blood pressure, mmHg^a^	147.9±24.9	151.4±22.7		−1.8	0.065
Diastolic blood pressure, mmHg^a^	84.0±12.7	84.0±12.0		0.026	0.979
Platelet count (10^9^/l)^a^	237.345±78.032	245.263±78.838		−1.229	0.220
International normalized ratio^a^	1.029±0.101	1.020±0.118		0.884	0.377
Plasma fibrinogen (g/l)^a^	3.460±0.859	3.871±1.045		−5.124	<0.001
Total cholesterol (mmol/l)^a^	4.862±1.199	5.023±1.206		−1.632	0.103
Triglyceride (mmol/l)^a^	1.642±0.996	1.753±1.140		−1.27	0.204
HDL (mmol/l)^a^	1.191±0.447	1.116±0.491		1.942	0.053
LDL (mmol/l)^a^	2.715±1.011	2.846±0.990		−1.598	0.111
HLR^a^	0.520±0.408	0.444±0.252		2.686	0.008
Fasting blood glucose (mmol/l)^a^	6.000±2.149	6.846±3.188		−3.874	<0.001
Glycated hemoglobin (%)^a^	6.415±1.837	7.009±1.985		−3.115	0.002
Homocysteine (μmol/l)^a^	14.084±4.669	14.992±6.561		−1.615	0.107

a Mean ± SD.

HDL, high-density lipoprotein; LDL, low-density lipoprotein; HLR, HDL/LDL ratio.

**Table III:** Association between potential risk factors and the severity of stroke.

Risk factor	No stroke	Stroke with large artery atherosclerosis	Stroke with small artery atherosclerosis	Total	Test statistic	P-value
Gender, n (%)						
Male	113 (64.6)	154 (70.0)	109 (73.6)	376 (69.2)	3.201	0.202
Female	62 (35.4)	66 (30.0)	39 (26.4)	167 (30.8)		
Diabetes, n (%)						
No	140 (80.0)	131 (59.5)	109 (73.6)	380 (70.0)	20.714	<0.001
Yes	35 (20.0)	89 (40.5)	39 (26.4)	163 (30.0)		
Hypertension, n (%)						
No	59 (33.7)	34 (15.5)	19 (12.8)	112 (20.6)	27.388	<0.001
Yes	116 (66.3)	186 (84.5)	129 (87.2)	431 (79.4)		
Hyperlipidemia, n (%)						
No	104 (59.4)	88 (40.2)	75 (50.7)	267 (49.3)	14.578	0.001
Yes	71 (40.6)	131 (59.8)	73 (49.3)	275 (50.7)		
Smoking, n (%)						
No	128 (73.1)	119 (54.1)	90 (60.8)	337 (62.1)	15.161	0.001
Yes	47 (26.9)	101 (45.9)	58 (39.2)	206 (37.9)		
Wine consumption, n (%)						
No	167 (95.4)	175 (79.5)	126 (85.1)	468 (86.2)	20.845	<0.01
Yes	8 (4.6)	45 (20.5)	22 (14.9)	75 (13.6)		
Age, years^a^	61.7±11.3	65.5±11.1	62.6±11.6		6.2	0.002
Systolic blood pressure, mmHg^a^	140.7±21.4	152.54±22.9	157.3±23.0		24.1	<0.01
Diastolic blood pressure, mmHg^a^	79.6±9.8	85.7±12.4	88.2±12.7		27.5	<0.01
Platelet count (10^9^/l)^a^	233.751±72.418	244.735±76.258	247.944±83.259		1.559	0.211
International normalized ratio^a^	1.031±0.094	1.013±0.094	1.011±0.087		2.097	0.124
Plasma fibrinogen (g/l)^a^	3.368±0.879	3.984±1.103	3.659±0.835		17.363	<0.001
Total cholesterol (mmol/l)^a^	4.742±1.142	5.082±1.228	4.909±1.113		4.039	0.018
Triglyceride (mmol/l)^a^	1.677±1.236	1.762±1.071	1.719±0.972		0.291	0.748
HDL (mmol/l)^a^	1.192±0.407	1.114±0.566	1.142±0.463		1.189	0.305
LDL (mmol/l)^a^	2.542±0.907	2.936±0.997	2.796±0.984		7.966	<0.001
HLR^a^	0.542±0.357	0.418±0.212	0.498±0.454		8.833	<0.001
Fasting blood glucose (mmol/l)^a^	5.765±2.347	7.166±3.402	6.414±2.354		11.659	<0.001
Glycated hemoglobin (%)^a^	6.243±1.376	7.219±2.189	6.613±1.879		10.379	<0.001
Homocysteine (μmol/l)^a^	14.301±4.473	15.051±6.512	14.640±6.106		0.500	0.607

a Mean ± SD.

HDL, high-density lipoprotein; LDL, low-density lipoprotein; HLR, HDL/LDL ratio.

**Table IV:** Association between potential risk factors and progressive stroke.

	Progressive stroke				
				
Risk factor	No (n=368)	Yes (n=60)	Total	Test statistic	P-value
Gender, n (%)					
Male	262 (71.2)	43 (71.7)	305 (71.3)	0.006	0.940
Female	106 (28.8)	17 (28.3)	123 (28.7)		
Diabetes, n (%)					
No	248 (67.4)	40 (66.7)	288 (67.3)	0.012	0.912
Yes	120 (32.6)	20 (33.3)	140 (32.7)		
Hypertension, n (%)					
No	58 (15.8)	13 (21.7)	71 (16.6)	1.300	0.254
Yes	310 (84.2)	47 (78.3)	357 (83.4)		
Hyperlipidemia, n (%)					
No	179 (48.8)	21 (35.0)	200 (46.8)	3.929	0.047
Yes	188 (51.2)	39 (65.0)	227 (53.2)		
Smoking, n (%)					
No	217 (59.0)	29 (48.3)	246 (57.5)	2.387	0.122
Yes	151 (41.0)	31 (51.7)	182 (42.5)		
Wine consumption, n (%)					
No	318 (86.4)	37 (61.7)	355 (82.9)	22.331	<0.01
Yes	50 (13.6)	23 (38.3)	73 (17.1)		
Age, years^a^	63.5±11.1	68.8±12.0		−3.4	<0.01
Systolic blood pressure, mmHg^a^	153.6±24.1	152.8±19.0		0.3	0.766
Diastolic blood pressure, mmHg^a^	85.7±12.9	87.1±11.7		−0.8	0.432
Platelet count (10^9^/l)^a^	244.019±79.526	246.967±89.006		−0.261	0.794
International normalized ratio^a^	1.031±0.118	0.975±0.099		3.46	<0.01
Plasma fibrinogen (g/l)^a^	3.789±0.982	3.964±1.135		−1.234	0.218
Total cholesterol (mmol/l)^a^	4.980±1.229	5.278±1.148		−1.756	0.080
Triglyceride (mmol/l)^a^	1.706±1.022	1.686±0.923		0.14	0.889
HDL (mmol/l)^a^	1.121±0.355	1.204±0.998		−0.635	0.528
LDL (mmol/l)^a^	2.840±1.032	3.032±0.935		−1.349	0.178
HLR^a^	0.458±0.298	0.453±0.459		0.083	0.934
Fasting blood glucose (mmol/l)^a^	6.610±2.599	7.782±4.420		−1.997	0.050
Glycated hemoglobin (%)^a^	7.073±2.108	6.494±1.995		1.917	0.056
Homocysteine (μmol/l)^a^	14.847±6.432	13.731±5.049		1.429	0.156

a Mean ± SD.

HDL, high-density lipoprotein; LDL, low-density lipoprotein; HLR, HDL/LDL ratio.

**Table V:** Logistic regression analysis of factors affecting carotid atherosclerosis.

						95% CI of OR value	
						
Risk factor	B	SE	Wald	P-value	OR value	Lower limit	Upper limit
Hardening							
Intercept	−10.138	6.565	2.385	0.123			
Age	0.063	0.030	4.445	0.035	1.066	1.004	1.130
Systolic blood pressure	0.029	0.016	3.045	0.081	1.029	0.996	1.063
Platelet count	0.003	0.004	0.338	0.561	1.003	0.994	1.011
International normalized ratio	4.015	3.747	1.148	0.284	55.413	0.036	85,759.316
Plasma fibrinogen	0.386	0.406	0.901	0.342	1.471	0.663	3.260
Triglyceride	0.542	0.408	1.763	0.184	1.720	0.772	3.830
HDL	1.878	0.947	3.932	0.047	6.540	1.022	41.851
LDL	0.441	0.397	1.232	0.267	1.554	0.714	3.382
HLR	−0.269	1.235	0.048	0.827	0.764	0.068	8.601
Fasting blood glucose	0.197	0.172	1.314	0.252	1.218	0.870	1.705
Glycated hemoglobin	−0.929	0.363	6.546	0.011	0.395	0.194	0.805
Homocysteine	0.037	0.065	0.314	0.576	1.037	0.912	1.179
Gender	−0.319	0.781	0.167	0.683	0.727	0.157	3.356
Diabetes	2.322	1.161	3.999	0.046	10.195	1.047	99.254
Hypertension	0.887	0.742	1.428	0.232	2.427	0.567	10.390
Hyperlipidemia	0.640	0.806	0.629	0.428	1.896	0.390	9.203
Smoking	1.522	0.777	3.839	0.050	4.582	1.000	21.002
Wine consumption	−0.232	0.926	0.063	0.802	0.793	0.129	4.868
Plaque formation							
Intercept	−13.361	6.013	4.938	0.026			
Age	0.092	0.028	10.952	0.001	1.096	1.038	1.157
Systolic blood pressure	0.023	0.015	2.369	0.124	1.024	0.994	1.055
Platelet count	0.004	0.004	0.785	0.376	1.004	0.996	1.012
International normalized ratio	6.320	3.414	3.428	0.064	555.608	0.690	447,157.189
Plasma fibrinogen	0.454	0.377	1.449	0.229	1.574	0.752	3.296
Triglyceride	0.248	0.393	0.400	0.527	1.282	0.594	2.768
HDL	1.874	0.929	4.070	0.044	6.512	1.055	40.209
LDL	−0.220	0.377	0.341	0.559	0.802	0.383	1.681
HLR	−1.205	1.166	1.068	0.301	0.300	0.030	2.946
Fasting blood glucose	0.143	0.152	0.886	0.346	1.154	0.856	1.556
Glycated hemoglobin	−0.367	0.288	1.628	0.202	0.693	0.394	1.218
Homocysteine	0.056	0.061	0.844	0.358	1.058	0.938	1.193
Gender	0.092	0.702	0.017	0.896	1.096	0.277	4.341
Diabetes	2.163	1.092	3.921	0.048	8.700	1.022	77.035
Hypertension	1.147	0.635	3.269	0.071	3.150	0.908	10.927
Hyperlipidemia	1.236	0.730	2.864	0.091	3.441	0.822	14.393
Smoking	1.006	0.698	2.077	0.150	2.735	0.696	10.749
Wine consumption	0.337	0.840	0.160	0.689	01.400	0.270	7.267
Stenosis							
Intercept	−12.001	6.638	3.268	0.071			
Age	0.104	0.033	10.273	0.001	1.110	1.041	1.183
Systolic blood pressure	0.016	0.016	0.898	0.343	1.016	0.983	1.049
Platelet count	0.000	0.005	0.000	0.996	1.000	0.991	1.009
International normalized ratio	5.469	3.692	2.194	0.139	237.189	0.171	329,261.826
Plasma fibrinogen	0.539	0.403	1.787	0.181	1.713	0.778	3.773
Triglyceride	−0.067	0.461	0.021	0.884	0.935	0.379	2.307
HDL	1.833	1.100	2.780	0.095	6.256	0.725	53.975
LDL	−0.072	0.460	0.025	0.875	0.930	0.378	2.291
HLR	−2.148	1.809	1.410	0.235	0.117	0.003	4.047
Fasting blood glucose	0.133	0.165	0.647	0.421	1.142	0.827	1.577
Glycated hemoglobin	−0.406	0.317	1.637	0.201	0.666	0.358	1.241
Homocysteine	0.059	0.066	0.800	0.371	1.061	0.932	1.209
Gender	0.276	0.801	0.118	0.731	1.317	0.274	6.332
Diabetes	2.237	1.151	3.778	0.052	9.362	0.982	89.303
Hypertension	1.654	0.789	4.387	0.036	5.225	1.112	24.552
Hyperlipidemia	0.947	0.804	1.387	0.239	2.577	0.533	12.450
Smoking	1.449	0.767	3.570	0.059	4.259	0.947	19.142
Wine consumption	0.161	0.929	0.030	0.862	1.175	0.190	7.251

CI, confidence interval; SE, standard error; OR, odds ratio; HDL, high-density lipoprotein; LDL, low-density lipoprotein; HLR, HDL/LDL ratio.

**Table VI:** Logistic regression analysis of factors affecting cerebral artery stenosis (significant factors).

						95% CI of OR value	
						
Risk factor	B	SE	Wald	P-value	OR value	Lower limit	Upper limit
Age	0.030	0.012	6.174	0.013	1.031	1.006	1.056
Diabetes	1.054	0.295	12.772	0.000	2.869	1.609	5.133
Plasma fibrinogen	0.299	0.147	4.148	0.042	1.348	1.011	1.797
HLR	−0.925	0.420	4.841	0.028	0.396	0.174	0.904

HLR, high-density lipoprotein/low-density lipoprotein ratio; CI, confidence interval; OR, odds ratio; SE, standard error.

**Table VII:** Logistic regression analysis of factors affecting stroke.

						95% CI of OR value	
						
Risk factor	B	SE	Wald	P-value	OR value	Lower limit	Upper limit
Large artery atherosclerosis							
Intercept	−6.995	3.664	3.645	0.056			
Age	0.010	0.019	0.262	0.609	1.010	0.972	1.049
Systolic blood pressure	0.013	0.009	1.763	0.184	1.013	0.994	1.031
Platelet count	−0.001	0.003	0.153	0.696	0.999	0.994	1.004
International normalized ratio	−1.453	2.100	0.479	0.489	0.234	0.004	14.348
Plasma fibrinogen	0.356	0.235	2.294	0.130	1.427	0.901	2.261
Triglyceride	−0.048	0.208	0.053	0.817	0.953	0.635	1.432
HDL	0.021	0.750	0.001	0.978	1.021	0.235	4.441
LDL	0.381	0.411	0.858	0.354	1.464	0.654	3.279
HLR	−0.690	1.890	0.133	0.715	0.502	0.012	20.399
Fasting blood glucose	0.400	0.174	5.302	0.021	1.492	1.061	2.097
Glycated hemoglobin	0.308	0.259	1.419	0.234	1.361	0.819	2.261
Homocysteine	−0.020	0.038	0.271	0.602	0.981	0.910	1.056
Gender	0.296	0.479	0.383	0.536	1.345	0.526	3.439
Diabetes	0.111	0.552	0.040	0.841	1.117	0.379	3.294
Hypertension	0.293	0.530	0.306	0.580	1.341	0.474	3.789
Hyperlipidemia	0.505	0.454	1.239	0.266	1.657	0.681	4.034
Smoking	0.183	0.464	0.155	0.694	1.200	0.483	2.983
Wine consumption	1.635	0.728	5.041	0.025	5.127	1.231	21.358
Small artery occlusion							
Intercept	−5.180	3.822	1.837	0.175			
Age	−0.005	0.020	0.063	0.802	0.995	0.956	1.035
Systolic blood pressure	0.019	0.010	3.312	0.069	1.019	0.999	1.039
Platelet count	0.003	0.003	1.292	0.256	1.003	0.998	1.008
International normalized ratio	−3.342	2.372	1.985	0.159	0.035	0.000	3.697
Plasma fibrinogen	0.061	0.250	0.060	0.806	1.063	0.652	1.735
Triglyceride	−0.018	0.212	0.007	0.932	0.982	0.648	1.489
HDL	−1.230	0.926	1.763	0.184	0.292	0.048	1.796
LDL	0.803	0.422	3.626	0.057	2.232	0.977	5.100
HLR	2.486	1.812	1.881	0.170	12.010	0.344	418.996
Fasting blood glucose	0.361	0.180	4.022	0.045	1.434	1.008	2.041
Glycated hemoglobin	0.196	0.271	0.523	0.469	1.217	0.715	2.069
Homocysteine	−0.054	0.042	1.671	0.196	0.947	0.873	1.028
Gender	0.285	0.510	0.312	0.577	1.330	0.489	3.615
Diabetes	0.005	0.607	0.000	0.994	1.005	0.306	3.302
Hypertension	0.736	0.593	1.542	0.214	2.088	0.653	6.679
Hyperlipidemia	−0.128	0.495	0.067	0.796	0.880	0.333	2.322
Hyperlipidemia	0.068	0.501	0.018	0.892	1.070	0.401	2.855
Smoking	1.513	0.768	3.886	0.049	4.542	1.009	20.455

CI, confidence interval; OR, odds ratio; SE, standard error; HDL, high-density lipoprotein; LDL, low-density lipoprotein; HLR, HDL/LDL ratio.

**Table VIII:** Logistic regression analysis of factors affecting progressive stroke.

						95% CI of OR value	
						
Risk factor	B	SE	Wald	P-value	OR value	Lower limit	Upper limit
Age	0.067	0.018	14.401	<0.001	1.070	1.033	1.108
Wine consumption	1.724	0.400	18.570	0.001	5.608	2.560	12.286
International normalized ratio	−6.955	2.292	9.203	0.002	0.001	0.000	0.085
Fasting blood glucose	0.314	0.079	15.916	<0.001	1.369	1.173	1.598
Glycated hemoglobin	−0.553	0.158	12.290	<0.001	0.575	0.423	0.784

International normalized ratio and glycated hemoglobin were protective factors (P<0.05), fasting blood glucose, age and alcohol consumption were risk factors (P<0.05). In total factor model, only 57 cases occurred progressive stroke so the single factor was age, hyperlipidemia, drinking, international normalized ratio, fasting blood glucose, glycated hemoglobin (P=0.057). CI, confidence interval; OR, odds ratio; SE, standard error.

**Table IX:** Effect of fasting blood glucose and FIB classification on carotid atherosclerosis.

						95% CI of OR value	
						
Risk factor	B	SE	Wald	P-value	OR value	Lower limit	Upper limit
Hardening							
Intercept	−13.523	6.101	4.912	0.027			
Age	0.066	0.031	4.533	0.033	1.069	1.005	1.136
Systolic blood pressure	0.028	0.017	2.821	0.093	1.029	0.995	1.063
Platelet count	0.002	0.004	0.236	0.627	1.002	0.993	1.011
International normalized ratio	4.318	3.937	1.202	0.273	75.015	0.033	168,551.521
Triglyceride	0.732	0.445	2.708	0.100	2.079	0.870	4.971
HDL	1.812	1.011	3.215	0.073	6.124	0.845	44.394
LDL	0.551	0.413	1.780	0.182	1.735	0.772	3.898
HLR	−0.175	1.264	0.019	0.890	0.839	0.070	10.005
Glycated hemoglobin	−0.872	0.345	6.381	0.012	0.418	0.212	0.822
Homocysteine	0.025	0.065	0.150	0.698	1.026	0.902	1.166
Diabetes	2.883	1.210	5.681	0.017	17.875	1.669	191.421
Hypertension	0.906	0.801	1.280	0.258	2.474	0.515	11.888
Hyperlipidemia	0.283	0.835	0.115	0.735	1.327	0.258	6.816
Smoking	1.578	0.796	3.934	0.047	4.845	1.019	23.043
Wine consumption	−0.026	0.989	0.001	0.979	0.974	0.140	6.761
Gender	−0.274	0.816	0.113	0.737	0.760	0.154	3.760
Fasting blood glucose 1	0.313	1.013	0.096	0.757	1.368	0.188	9.965
Fasting blood glucose 2	0.133	1.014	0.017	0.896	1.142	0.157	8.328
Fasting blood glucose 3	−0.260	0.992	0.069	0.793	0.771	0.110	5.388
Plasma fibrinogen 1	−1.408	1.031	1.865	0.172	0.245	0.032	1.846
Plasma fibrinogen 2	0.242	0.978	0.061	0.805	1.273	0.187	8.654
Plasma fibrinogen 3	−0.125	0.936	0.018	0.893	0.882	0.141	5.523
Plaque formation							
Intercept	−17.606	5.621	9.811	0.002			
Age	0.094	0.029	10.782	0.001	1.099	1.039	1.162
Systolic blood pressure	0.023	0.016	2.254	0.133	1.024	0.993	1.055
Platelet count	0.004	0.004	0.795	0.373	1.004	0.996	1.012
International normalized ratio	6.246	3.601	3.009	0.083	516.125	0.444	599,451.170
Triglyceride	0.358	0.423	0.717	0.397	1.431	0.624	3.280
HDL	1.847	0.989	3.490	0.062	6.343	0.913	44.055
LDL	−0.133	0.394	0.113	0.736	0.876	0.405	1.896
HLR	−1.207	1.202	1.010	0.315	0.299	0.028	3.151
Glycated hemoglobin	−0.275	0.259	1.122	0.289	0.760	0.457	1.263
Homocysteine	0.054	0.061	0.769	0.381	1.055	0.936	1.190
Diabetes	2.713	1.146	5.609	0.018	15.076	1.596	142.377
Hypertension	1.384	0.692	3.998	0.046	3.991	1.028	15.500
Hyperlipidemia	0.877	0.753	1.355	0.244	2.403	0.549	10.518
Smoking	1.105	0.719	2.364	0.124	3.019	0.738	12.350
Wine consumption	0.727	0.892	0.664	0.415	2.069	0.360	11.890
Gender	0.033	0.737	0.002	0.964	1.034	0.244	4.385
Fasting blood glucose 1	0.582	0.915	0.404	0.525	1.790	0.298	10.766
Fasting blood glucose 2	0.422	0.916	0.212	0.645	1.525	0.253	9.172
Fasting blood glucose 3	−0.182	0.904	0.040	0.841	0.834	0.142	4.902
Plasma fibrinogen 1	−1.163	0.929	1.568	0.211	0.313	0.051	1.930
Plasma fibrinogen 2	0.262	0.893	0.086	0.769	1.300	0.226	7.476
Plasma fibrinogen 3	−0.938	0.879	1.137	0.286	0.392	0.070	2.194
Stenosis							
Intercept	−17.095	6.254	7.471	0.006			
Age	0.094	0.033	7.945	0.005	1.099	1.029	1.173
Systolic blood pressure	0.013	0.017	0.603	0.437	1.013	0.980	1.048
Platelet count	−0.001	0.005	0.041	0.839	0.999	0.990	1.008
International normalized ratio	6.102	3.870	2.487	0.115	446.693	0.227	878,531.134
Triglyceride	−0.010	0.480	0.000	0.984	0.990	0.387	2.535
HDL	1.555	1.182	1.729	0.189	4.733	0.466	48.022
LDL	−0.034	0.492	0.005	0.945	0.966	0.368	2.536
HLR	−1.891	1.908	0.982	0.322	0.151	0.004	6.353
Glycated hemoglobin	−0.157	0.288	0.295	0.587	0.855	0.486	1.504
Homocysteine	0.060	0.067	0.799	0.371	1.062	0.931	1.210
Diabetes	2.665	1.215	4.808	0.028	14.367	1.327	155.573
Hypertension	1.943	0.861	5.091	0.024	6.979	1.291	37.737
Hyperlipidemia	0.685	0.827	0.686	0.407	1.985	0.392	10.048
Smoking	1.584	0.787	4.055	0.044	4.874	1.043	22.773
Wine consumption	0.555	0.974	0.326	0.568	1.743	0.259	11.746
Gender	0.161	0.846	0.036	0.849	1.174	0.224	6.161
Fasting blood glucose 1	1.193	1.063	1.260	0.262	3.298	0.411	26.501
Fasting blood glucose 2	0.979	1.089	0.808	0.369	2.663	0.315	22.524
Fasting blood glucose 3	1.366	1.009	1.835	0.176	3.921	0.543	28.311
Plasma fibrinogen 1	−1.024	0.994	1.061	0.303	0.359	0.051	2.521
Plasma fibrinogen 2	−1.151	1.053	1.194	0.275	0.316	0.040	2.492
Plasma fibrinogen 3	−0.871	0.936	0.866	0.352	0.418	0.067	2.620

FIB, fibrinogen; CI, confidence interval; OR, odds ratio; SE, standard error; HDL, high-density lipoprotein; LDL, low-density lipoprotein; HLR, HDL/LDL ratio.

**Table X:** Effect of fasting blood glucose and FIB classification on cerebral artery stenosis.

						95% CI of OR value	
						
Factors	B	SE	Wald	P-value	OR value	Lower limit	Upper limit
Gender	0.033	0.361	0.009	0.926	1.034	0.509	2.100
Age	0.031	0.014	4.796	0.029	1.032	1.003	1.061
Diabetes	0.946	0.415	5.186	0.023	2.574	1.141	5.809
Hypertension	0.185	0.404	0.210	0.647	1.203	0.545	2.659
Hyperlipidemia	0.585	0.352	2.759	0.097	1.794	0.900	3.576
Smoking	0.265	0.346	0.586	0.444	1.303	0.662	2.565
Wine consumption	0.239	0.437	0.300	0.584	1.270	0.540	2.990
Systolic blood pressure	0.002	0.007	0.110	0.740	1.002	0.989	1.015
Platelet count	0.000	0.002	0.035	0.852	1.000	0.997	1.004
International normalized ratio	−0.400	1.450	0.076	0.783	0.671	0.039	11.492
Triglyceride	−0.142	0.162	0.772	0.380	0.868	0.632	1.191
HDL	−0.006	0.345	0.000	0.985	0.994	0.505	1.953
LDL	−0.055	0.203	0.074	0.785	0.946	0.636	1.408
HLR	−0.788	0.666	1.403	0.236	0.455	0.123	1.676
Glycated hemoglobin	0.009	0.128	0.005	0.946	1.009	0.785	1.295
Homocysteine	0.033	0.029	1.255	0.263	1.033	0.976	1.094
Fasting blood glucose			1.497	0.683			
Fasting blood glucose 1	−0.480	0.470	1.040	0.308	0.619	0.246	1.556
Fasting blood glucose 2	−0.276	0.477	0.335	0.563	0.759	0.298	1.932
Fasting blood glucose 3	−0.060	0.457	0.018	0.895	0.941	0.385	2.304
Plasma fibrinogen			1.967	0.579			
Plasma fibrinogen 1	−0.269	0.415	0.421	0.516	0.764	0.339	1.723
Plasma fibrinogen 2	−0.427	0.402	1.129	0.288	0.653	0.297	1.434
Plasma fibrinogen 3	0.058	0.398	0.022	0.883	1.060	0.486	2.311

HDL, high-density lipoprotein; LDL, low-density lipoprotein; HLR, HDL/LDL ratio; CI, confidence interval; OR, odds ratio; SE, standard error.
